# Impaired H-Reflex Gain during Postural Loaded Locomotion in Individuals Post-Stroke

**DOI:** 10.1371/journal.pone.0144007

**Published:** 2015-12-02

**Authors:** Jing Nong Liang, David A. Brown

**Affiliations:** 1 Department of Physical Therapy and Human Movement Sciences, Feinberg School of Medicine, Northwestern University, Chicago, Illinois, United States of America; 2 Interdepartmental Neuroscience Program, Feinberg School of Medicine, Northwestern University, Chicago Illinois, United States of America; 3 Department of Physical Therapy, University of Nevada Las Vegas, Las Vegas, Nevada, United States of America; 4 Department of Physical Therapy, School of Health Related Professions, University of Alabama at Birmingham, Birmingham, Alabama, United States of America; Duke University, UNITED STATES

## Abstract

**Objective:**

Successful execution of upright locomotion requires coordinated interaction between controllers for locomotion and posture. Our earlier research supported this model in the non-impaired and found impaired interaction in the post-stroke nervous system during locomotion. In this study, we sought to examine the role of the Ia afferent spinal loop, via the H-reflex response, under postural influence during a locomotor task. We tested the hypothesis that the ability to increase stretch reflex gain in response to postural loads during locomotion would be reduced post-stroke.

**Methods:**

Fifteen individuals with chronic post-stroke hemiparesis and 13 non-impaired controls pedaled on a motorized cycle ergometer with specialized backboard support system under (1) seated supported, and (2) non-seated postural-loaded conditions, generating matched pedal force outputs of two levels. H-reflexes were elicited at 90°crank angle.

**Results:**

We observed increased H-reflex gain with postural influence in non-impaired individuals, but a lack of increase in individuals post-stroke. Furthermore, we observed decreased H-reflex gain at higher postural loads in the stroke-impaired group.

**Conclusion:**

These findings suggest an impaired Ia afferent pathway potentially underlies the defects in the interaction between postural and locomotor control post-stroke and may explain reduced ability of paretic limb support during locomotor weight-bearing in individuals post-stroke.

**Significance:**

These results support the judicious use of bodyweight support training when first helping individuals post-stroke to regain locomotor pattern generation and weight-bearing capability.

## Introduction

Limb stiffness regulation is a function of reflex, intrinsic and passive components and is modulated relative to the phase in locomotor tasks, such as walking [1–5]. During stance phase, motor output must be coordinated to support body weight, respond to perturbations, and prepare for force production during the propulsive phase of walking. The H-reflex is a commonly used electrophysiological test to evaluate the spinal circuitry, elicited by electrically stimulating the afferent nerve, and is commonly used as a tool to quantify excitatory behavior of the monosynaptic group Ia afferent volleys in the spinal cord circuitry [6]. In earlier studies examining the Ia afferent spinal loop, common measured variables include peak-to-peak H-reflex amplitudes under matched background muscle activity[7, 8], H-reflex gain from the slope relationship between H-reflex amplitude and EMG amplitude across a range of EMG levels [5, 9], and H-reflex gain defined as the ratio of H-reflex amplitude to average background muscle activity [10, 11] used as functional measures of reflex modulation. In the non-impaired nervous system, during walking, the soleus (SOL) H-reflex amplitude increases in a ramp-like fashion in parallel with SOL muscle activity during the stance/loading phase, and strongly suppressed during the swing/non-loading phase [3–5, 12], contributing functionally to force production, load compensation, and thus body weight support during stance, and assist with propulsion [13, 14]. In the neurologically impaired system, loss of this phase-dependent modulation has been reported [15, 16]. Additionally, excitability of spinal reflex pathways is also dependent upon the magnitude of effort [17–19] and postural conditions [20–23]. However, under comparable postural conditions, earlier studies using various protocols reported inconsistent findings regarding the effects of limb loading on H-reflex amplitude [24–31].

Due to difficulties in elucidating the postural and locomotor components in a locomotor task, limited research has examined the influence of different magnitudes of postural loads on locomotor output in non-impaired nervous systems, and even more limited in stroke-impaired systems. Therefore, we have engaged in a series of pedaling experiments to study locomotor control in isolation of postural control mechanisms and systematically add postural influence, so as to examine the neural control mechanisms of post-stroke locomotion with and without the confounding effects of posture, within a mechanically-constrained locomotor context.

Previously, we found that when pedaling under different levels of weight-bearing versus under different effort levels, individuals post-stroke exhibited reduction in coordination control which worsened with increased weight-bearing but not with increased effort [32]. Furthermore, during a non-postural related locomotor task, the stroke-impaired nervous system had the ability to direct foot forces appropriately during a skilled, mechanically-constrained seated pedaling locomotor task [33, 34]. However, the ability to appropriately direct foot forces was observed to be impaired in the stroke-impaired nervous system when postural control involvement was added during locomotion, specifically, we observed inappropriate forward directed shear forces that were generated as a result of inappropriate paretic leg extensor activity [33].

In this current study, we sought to compare the role of the Ia afferent spinal loop under two conditions during a locomotor pedaling task: (1) postural loaded pedaling, and (2) non-postural loaded pedaling, in the non-impaired and in the post-stroke nervous system. Based on the findings of our earlier studies [33, 34], we found that foot force directional control during non-postural loaded seated pedaling were well regulated in people post-stroke, but became impaired when postural loads were imposed on the pedaling task. This poor control was further exaggerated with greater postural loads. Thus, with this study, we expected to see impaired modulation of H-reflex gain underlying the impaired foot force directional control when postural loads were imposed on the pedaling task, and worsened with increased postural loads. Addition of postural load would require higher reflex stiffness to compensate to prevent collapse during pedaling, therefore, we hypothesized that, with postural involvement during a locomotor task in non-impaired individuals, H-reflex gain (defined in this study as the ratio of H-reflex amplitude to SOL background activity) would increase during non-seated postural-loaded pedaling, to provide against stretch perturbations that might result in collapse. However, in individuals post-stroke we hypothesized that H-reflex gain measured from the paretic legs would have a lack of increase or even a decrease, which might explain the reduction in limb stiffness under conditions of loading during upright locomotion post-stroke. Finally, we hypothesized that, with higher postural load levels, we would expect further reduced H-reflex gain in individuals post-stroke. A subset of the data collected during this experiment has been reported in an earlier paper [33].

## Methods

### Subjects

Fifteen individuals (age = 58.8 ± 6.6 (mean ± SD) years), who had sustained a single, unilateral, cortical or subcortical stroke, more than 12 months postictus (134.5 ± 55.7 months) before the study and had residual lower limb hemiparesis without lower limb contractures participated in this study. Thirteen age-similar non-neurologically impaired individuals (age = 55.4 ± 11.3 years) were recruited as controls. Ambulatory ability of individuals post-stroke ranged from independent ambulation without assistive devices to independent ambulation with cane/quad-cane/ankle-foot orthosis. As described in our earlier paper [33], participants were excluded from the experiment if they had other neurological conditions, cognitive or affective disorders, expressive or receptive aphasia, severe concurrent medical problems such as severe cardiac disease, history of poorly controlled brittle diabetes, active cancer, etc., orthopedic conditions affecting the legs, history of hip or knee replacement, or peripheral nerve injury in the lower limb. Each participant received written and verbal information about the experiment procedures before giving written consent. The protocol was approved by the Institutional Review Board at Northwestern University.

### Experimental Apparatus

**Fig 1:**
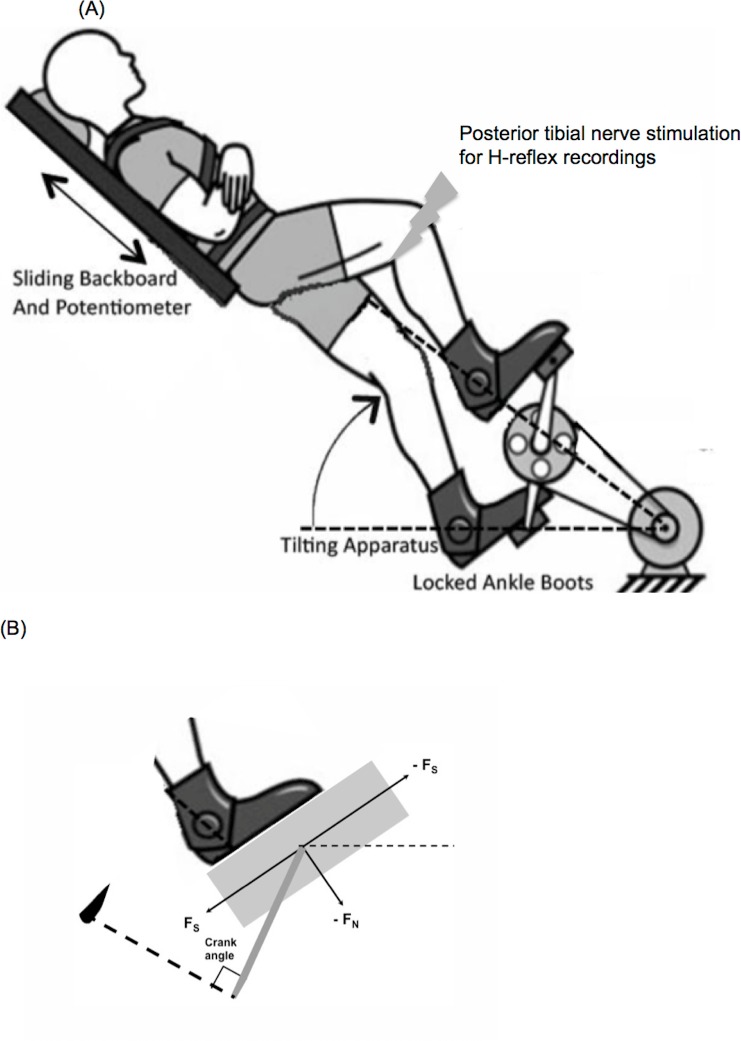
Pedaling apparatus. (A) Illustration of the custom-made cycle ergometer with instrumented pedals, a seat with backrest and a motor-driven crank. The backrest was locked in place to allow a locomotor task with minimal demands for postural control (Seated, non-postural loaded pedaling), or was unlocked to slide to add postural component to the locomotor task (Non-seated, postural loaded pedaling). (B) Pedal forces in the pedal coordinates system.

We used a custom-made, split-crank, cycle ergometer (Fig 1A) with instrumented pedals, a seat with backrest, and a motor-driven crank in this study. Participants were seated on the seat with the torso stabilized against the backrest to maintain constant hip position. Optical encoders (BEI model EX116-1024-2), one at each pedal spindle and one coupled to the right crank, provided measurements of the pedal angles and the crank position with ±0.3° accuracy. Force transducers in each pedal measured the three-dimensional foot/pedal force vector (Delta 660, ATI-IA Inc, Garner, NC). A custom-made boot with Velcro straps was attached to each pedal to minimize ankle movement during the pedaling tasks. Pedaling velocity was controlled by an electric motor (12:1 gear reducer, 3.7hp; model MT506B1-S1C1, Kollmorgen, Radford, VA) and was kept constant at 40 revolutions/minute (rpm) for all subjects during the experiment.

### Electromyography (EMG) Recordings

EMG activities from the SOL and vastus medialis (VM) muscles were recorded from the test leg using bipolar silver surface electrodes (DelSys, 1cm length, 1mm wide, 1cm inter-electrode distance). EMG signals were amplified with a gain of 10 at the electrode site before remote differential amplification (CMRR: 92dB, gain range 100–1000 times, frequency response 20-450Hz) and were low pass filtered (custom-designed filter, 500Hz cutoff). The signals from the optical encoders and force-transducers were converted from digital to analog with a D/A converter module before sampling. All signals were then sampled at 2000Hz via a 12-bit A/D converter (National Instruments) and custom LabView software.

### SOL H-reflex elicitation and recording protocol

SOL H-reflexes and M-waves were elicited using a constant current stimulator and isolation unit (DS7A, Digitimer Ltd, Welwyn Garden City, UK) with a square pulse stimuli of 1ms duration, a current range of 50μA~200mA and total output capability of 400V. Following standard skin preparation procedures, bipolar stimulating electrodes were placed over the posterior tibial nerve at the popliteal fossa. The electrodes were stabilized using adhesive tape and velcro to ensure minimal movement during pedaling. Stimulations were triggered by a digital pulse from a data-acquisition board that was under feedback control from the optical encoder signaling crank position. For each participant, in the seated non-postural loaded position with backboard tilted at 40° and test leg positioned at 90° crank position, we elicited the SOL H-reflex recruitment curve by increasing the stimulus intensity at small increments until a maximal H-reflex was obtained followed by attainment of a maximum M wave. From the recruitment curve, we identified the intensity needed to elicit a SOL H-reflex on the ascending limb of the H-reflex recruitment curve and is accompanied by a M wave, which was kept constant for all trials to ensure consistency of stimulations. For each condition, approximately 20 stimuli were delivered at 90° (from top dead center) crank position, in the middle of the downstroke. The reflex stimulations were delivered at an inter-stimulus interval of at least 8 seconds to minimize effects of post-activation depression [35].

### Experimental paradigm

While seated on the bike seat, and with the crank fixed at 90° in the middle of the downstroke (crank angle defined relative to top-dead-center), participants were instructed to generate 3 maximal force efforts with their test leg, which was the paretic leg for individuals post-stroke and the dominant leg for non-impaired individuals. The pedal normal force vector (F_N_) magnitude generated from the maximal pushes (max F_N_) was averaged across the 3 trials.

Each participant pedaled along with the motor-driven crank moving at 40 rpm under: (1) seated, non-postural loaded condition, and (2) non-seated, postural loaded condition. For each condition, there were 2 effort levels: (1) low effort level (30% max F_N_) and (2) high effort level (50% max F_N_), as described in our earlier paper [33].

For the seated, non-postural loaded condition (S), participants, while seated on the bike seat, were instructed to assist the motor actively by pedaling along with the moving crank in the forward direction, generating a target F_N_ of 30% and 50% max F_N_ with visual feedback. For all seated pedaling conditions, the tilt angle of the backboard was kept at 40° from horizontal via a hydraulic tilt mechanism. Bar graphs with real-time pedal F_N_ were displayed as visual feedback on a monitor. Each pedaling trial lasted 30 seconds with breaks in between to avoid effects of fatigue.

For the non-seated, postural loaded condition (NS), the bike seat was lowered to the minimal height possible, so it would not interfere as the participant stood up to pedal without sitting on the seat, and the backboard was unlocked to yield one extra degree of freedom (Fig 1A). The tilt angle of the backboard was adjusted to yield a peak F_N_ of 30% and 50% max F_N_ that matched the target 30% and 50% max F_N_ generated during the seated pedaling conditions. This determination of the tilt angle was done on a subject-by-subject basis, based upon their particular body weights. Participants were instructed to actively push away from the seat, supporting their own body weight, while pedaling along with the moving crank. Trials were repeated until 20 H-reflexes were collected for each condition.

### Data Processing and Analysis

All forces, EMG and H-reflex data were processed and analyzed using custom MATLAB programs. Force profiles were expressed in the pedal coordinates system where shear pedal forces (F_S_) with negative value indicates anteriorly-directed shear and positive values indicate posteriorly-directed shear, and normal forces with negative values indicate forces directed downwards (Fig 1B). For each task and effort level, we averaged the peak F_N_ achieved and compared the means using independent t-test, to ensure we were able to match the target F_N_ across conditions.

For each condition, the peak-to-peak amplitude of SOL H-reflexes, and its corresponding M-waves was calculated using a custom Matlab analysis program and averaged. To ensure consistency of the stimulation, if a trial had a M-wave that fell outside the desired range, that trial was not included in the analysis. For analysis of the background EMG, we used a custom Matlab analysis program where, for each trial, EMG activity over the duration of 100ms prior to the electrical stimulus were rectified and smoothed with a fourth-order, zero-lag, low-pass Butterworth filter with a cutoff frequency of 25Hz. Then the EMG activity 100ms prior to the delivery of the electrical stimulus was integrated. The EMG activity over the 100ms was used to normalize the peak-to-peak amplitude of the H-reflex and expressed as H/EMG ratio, indicative of the H-reflex gain.

The mean corresponding control M-waves were compared across conditions using repeated measures analysis of variance (ANOVA). Two-way repeated measures ANOVA was used to analyze the mean H/EMG ratio, H-reflex amplitude and background EMG for each group separately. The independent variables included load (S and NS) and effort (30% and 50%). If interaction effects were observed, we performed a Bonferroni post-hoc analysis. A p-value less than or equal to 0.05 was considered statistically significant.

## Results

### Experimental control variables

To support the credibility of the results describing changes in H-reflex gain during the various experimental conditions, we first report on the validity of our experimental controls. We present results that describe our ability to control for force levels, M-wave stability, and we report the SOL and VM background EMG as an indication of background excitability under the four different experimental conditions.

As reported previously [33], we were successful in matching F_N_ generated during the non-seated postural loaded conditions with the targeted F_N_ that was generated during the seated conditions by adjusting the tilt angle of the backboard (15.20±2.24(SD) in stroke versus 17.23±2.49 in non-impaired at 30% effort; 20.40±2.95 in stroke versus 26.69±4.13 in non-impaired at 50% effort). Since F_S_ magnitude is partially dependent upon overall push effort and foot orientation relative to the pedal [34], similar F_N_ values, generated when the foot was secured in a neutral ankle position, allowed us to make valid comparisons of effort under seated and non-seated conditions within and between each group. Furthermore, to control for the sensitivity of the reflex to muscle length, the lower limbs were secured in locked boots that minimize ankle movement during the pedaling task. We also examined the pedal angle trajectories to ensure that the ankle joint was well constrained in a neutral posture by our custom-designed boot during pedaling [33].

In our comparison of reflex data, we adjusted the stimulation intensity such that the corresponding M-wave of the test H-reflex was in the desired range, to ensure consistency of stimulus intensity for all subjects under all experimental conditions. For the corresponding control M-waves of the tested H-reflex amplitudes, we did not observe a statistically significant difference across conditions within each subject (p>0.05).

**Fig 2:**
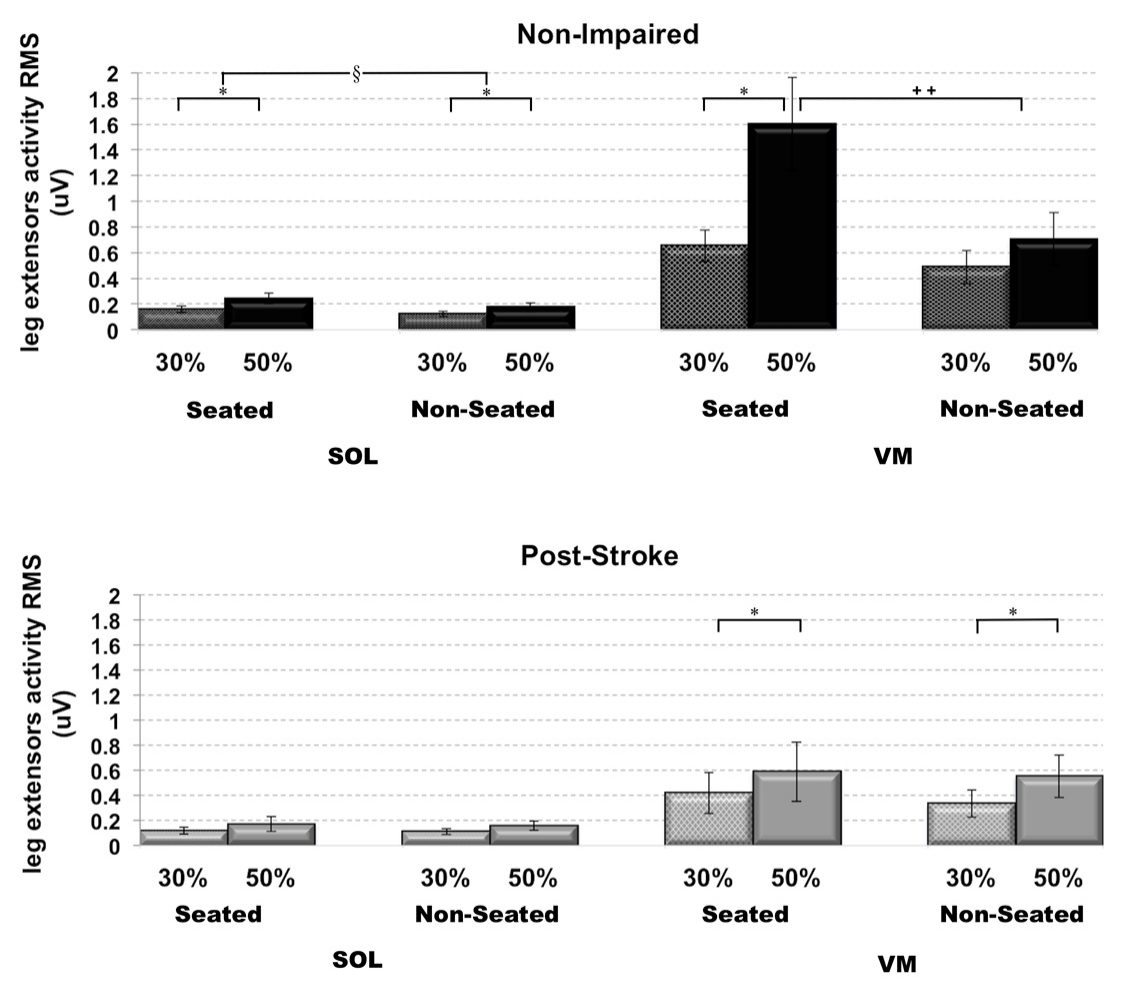
Inappropriate paretic extensor activity during postural loaded cycling. Changes in Soleus (SOL) and Vastus Medialis (VM) EMG activity in non-impaired (top, black) and post-stroke (bottom, grey) individuals. * indicate between effort and § indicate between loading conditions. **++** indicate difference between loading conditions at high effort levels only. Statistical significance at p<0.05 level.

For non-impaired individuals, when comparing SOL background EMG 100ms prior to the electrical stimulus, using two-way repeated measures ANOVA, we observed statistically significant main effects for both load (F_(1,12)_ = 5.96, p = 0.03) and effort levels (F_(1,12)_ = 17.70, p = 0.001), such that the SOL background EMG was lower during the non-seated (0.15±0.03) compared to the seated (0.20±0.03) pedaling conditions, and was greater during high effort (0.21±0.04) compared to low effort (0.14±0.02) conditions. In individuals post-stroke, we did not observe a change in paretic SOL background EMG 100ms prior to electrical stimulus in both seated (0.15±0.04) versus non-seated (0.14±0.03) pedaling (F_(1,14)_ = 0.17, p>0.05), and low effort (0.12±0.03) versus high effort (0.17±0.05) conditions (F_(1,14)_ = 4.16, p>0.05) (Fig 2).

For VM EMG activity, in non-impaired individuals, a two-way repeated measures ANOVA yielded a statistically significant interaction effect (F_(1,12)_ = 12.74, p = 0.004), such that the VM activity was greater in the seated (1.60±0.36) versus non-seated (0.71±0.20) at high effort conditions only, and was greater during high effort (1.60±0.36) compared to low effort (0.65±0.12) in the seated pealing conditions only. In individuals post-stroke, we observed a statistically significant main effect for effort levels only (F_(1,14)_ = 7.61, p = 0.02), such that the paretic VM EMG activity was increased for high effort (0.57±0.20) versus low effort (0.38±0.13) conditions, but unchanged for seated (0.50±0.20) versus non-seated (0.45±0.14) pedaling conditions (F_(1,14)_ = 0.38, p>0.05) (Fig 2).

### Effects of postural loading on H-reflex gain

**Fig 3:**
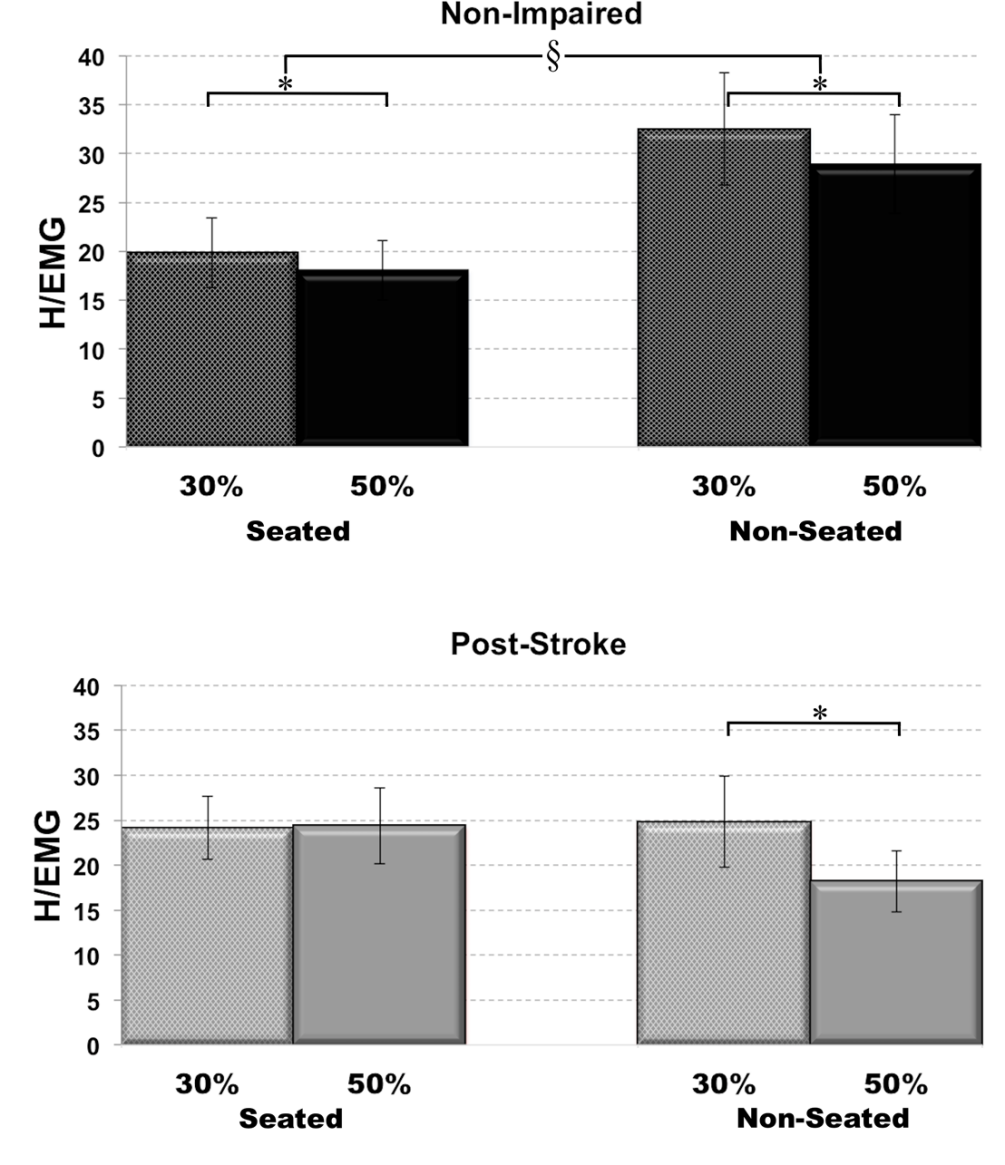
Impaired H-reflex gain during postural loaded cycling post-stroke. H-reflex gain expressed in H/EMG ratio in non-impaired (top, black) and post-stroke (bottom, grey) individuals. * indicate between effort and § indicate between loading conditions. Statistical significance at p<0.05 level.

In order to account for the different SOL background EMG under the different pedaling conditions, we expressed H-reflex amplitude as a ratio of the H-reflex amplitude to background SOL EMG activity, as an indication of the H-reflex gain (H/EMG ratio). When comparing seated versus non-seated pedaling conditions, H-reflex gain was increased in non-impaired individuals, but did not change in individuals post-stroke. For non-impaired individuals, two-way repeated measures ANOVA yielded a statistically significant main effect for seated versus non-seated condition (F_(1,12)_ = 15.11, p = 0.002), such that the average H/EMG ratio was significantly higher for non-seated pedaling (30.74±5.33) when compared to seated pedaling (18.96±3.21). The increase in H/EMG ratio with postural loading for non-impaired individuals was due to a decrease in SOL background EMG, rather than an increase in H-reflex amplitude. However, for individuals post-stroke, a two-way repeated measures ANOVA yielded a statistically significant interaction effect (F_(1,14)_ = 173.19, p = 0.048), but post hoc analysis yielded no significant difference in H/EMG ratio in the paretic leg between seated (24.26±3.76) and non-seated (21.52±4.11) pedaling conditions. This lack of H/EMG ratio difference for seated vs non-seated pedaling was reflected in the fact that we did not observe differences in both H-reflex amplitude and SOL background EMG values (Fig 3).

### Effect of effort on H-reflex gain

When comparing low versus high effort conditions, in non-impaired individuals H-reflex gain was decreased with higher effort, but was decreased in individuals post-stroke only during non-seated postural loaded conditions. For non-impaired individuals, two-way repeated measures ANOVA yielded a statistically significant main effect for low versus high effort conditions (F_(1,12)_ = 5.26, p = 0.041), such that average H/EMG ratio was significantly lower for high effort (23.51±3.84) when compared to low effort (26.19±4.47) levels, regardless of postural loading conditions. The decrease in H/EMG ratio with higher effort for non-impaired individuals was due to an increase in H-reflex amplitude accompanied by even greater increase in SOL background EMG at higher effort levels. For individuals post-stroke, a two-way repeated measures ANOVA yielded a statistically significant interaction effect (F_(1,14)_ = 173.19, p = 0.048), such that the H/EMG ratio was lower at high effort (18.22±3.42) versus low effort (24.82±5.06) levels during non-seated conditions only. This observed difference was due to an increased SOL EMG with no change in H-reflex amplitudes with increased effort levels (Fig 3).

## Discussion

In this study, we examined the role of interactions between postural control and the Ia afferent pathway under locomotor pedaling conditions with different postural control and force level demands in post-stroke and in non-impaired individuals. We found that, with postural loaded pedaling, H-reflex gain was appropriately increased in non-impaired individuals, but not in individuals post-stroke. The results supported our main hypothesis since greater postural effort requires higher reflex stiffness to compensate for the demands of body-weight support. However, due to poor reflex modulation, we did not expect to observe an appropriate reflex gain increase in the stroke-impaired system. With respect to force demand levels during postural loaded pedaling, we observed further decreased H-reflex gain in individuals post-stroke, which supported our hypothesis that with higher effort under postural loaded conditions, impaired reflex modulation would be worsened.

### Pedaling paradigm used to investigate postural load versus effort load during a locomotor-like task

In the past, we have successfully used the pedaling paradigm as a controlled model to study locomotor control [36–43]. To the best of our knowledge, this line of research done in our lab [32, 33] is the first to use a mechanically-constrained paradigm to make comparisons of a locomotor task with minimal postural control demands against a locomotor task with different levels of postural control under similar mechanical task demands, thereby allowing us to systematically investigate the interaction of control for posture and for locomotion in stroke-impaired and non-impaired nervous systems.

This novel pedaling paradigm allowed us to control variables that were very difficult to control in studies using body-weight support systems during upright walking, where the nature of the suspension could not constrain postural control mechanisms from interacting with locomotor control. The locomotor task was designed such that participants performed biomechanically controlled locomotor tasks, under posturally challenged pedaling conditions while they generated mechanical output (normal pedal force values) at fixed speeds that was comparable to pedaling conditions without postural challenge, thus allowing us to monitor the strategies that the nervous system adopts when postural conditions were imposed [33]. In the postural loaded conditions, the backboard orientation was adjusted to yield matching F_N_ generated during seated non-postural loaded conditions for each individual, and we recognize that H-reflexes can be sensitive to body orientations via tonic labyrinthine reflex influences [44]. However, the differences in tilt angle on average, between the post-stroke and non-impaired individuals were relatively small.

### Impaired interaction of postural control and locomotor control post-stroke

In the non-impaired nervous system, it has been proposed that movement and posture are controlled independently, and interact to act on locomotor networks to produce coordinated motor output [45]. Our earlier findings supported this model in the non-impaired system and also suggested that, following stroke, this interaction is impaired [33]. Our current results further suggest that the impaired interaction is expressed onto the Ia reflex pathway that regulates reflex stiffness during postural tasks.

With respect to leg extensor activity, we observed less non-impaired SOL and VM activity with postural loading, whereas the paretic leg extensors activity remained relatively unchanged, supporting our previous observations [33]. Reduced SOL activity during postural loaded locomotion was expected as mass of the trunk provided much of the force during the downstroke, and the ankle movement was minimized by the boot, so that little SOL activity was required to propel the crank. Similarly with VM muscle activity, assistance from gravitational forces on both the weight of the limb and the center of mass aided in the crank propulsion, and thus required less VM activity to accomplish the task.

During postural loaded pedaling, with non-impaired individuals, we observed increased H-reflex gain with postural loading, suggesting that greater limb reflex stiffness allowed for support to prevent collapse during postural loaded pedaling. H-reflex gain and H/M ratio has previously been reported to increase with body weight loads during standing [27, 46]. Even under stationary conditions, there may be small variations in the SOL muscle length, which would alter the Ia afferent input into the muscle, then indirectly contributing to a change in H-reflex amplitude. An underlying change in presynaptic inhibition on the SOL Ia afferent has been suggested as a possible mechanism [46, 47]. With increased postural loads, we observed no change in H-reflex gain in the non-impaired legs, reflected in the proportional increase in SOL background EMG and H-reflex amplitude. In general, in the non-impaired nervous system, for a given task, with greater background EMG, there were higher H-reflex amplitudes, due to the increase in excitability of the motoneuron pool at the same reflex gain [6, 18, 48]. With non-impaired individuals, similar results of unchanged H-reflex gains with alterations in body weight support levels have also been reported in earlier studies using body-weight supported system [49, 50], as well as under simulated reduced gravity conditions during walking and running tasks [9]. However, the linear increase in H-reflex amplitude with increase in background EMG can plateau at higher levels of EMG activity [51], potentially suggesting a limitation of using the H-reflex to assess the spinal circuitry over the entire range of excitatory drive. This saturation effect where the H-reflex amplitude is less influenced by background EMG activity at higher levels of EMG likely accounts for the observed decrease in H/EMG ratio at higher effort levels in this study.

However, in the paretic legs, during postural loaded pedaling, not only did we observe a lack of increase in the paretic H-reflex gain, but also the gain was further decreased at higher postural load efforts. This impaired reflex gain modulation may contribute to lack of weight-bearing capability during locomotor tasks post-stroke. At higher postural loads, the H-reflex gain was decreased, primarily as a result of greater paretic SOL background EMG but unchanged H-reflex amplitude. The lack of increase in the paretic SOL H-reflex amplitude with postural loads would indicate a possible deficit in the spinal mediated pathway in the support for postural loading in the stroke-impaired nervous system. Clinically, individuals with post-stroke hemiplegia are prescribed walking aids and body-weight supported treadmill training to improve functional measures [52–56]. This reliance on weight-supporting assistance indicates an impaired ability of the stroke-impaired individuals to bear postural loads. The positive functional effects of unweighing the paretic upper limb has been extensively studied [57–59], and in the paretic lower extremities, improved coordination control have been observed with reduced weight-bearing during pedaling [32]. In contrast to our observations with non-impaired individuals, the underlying mechanism that interferes with full weight bearing during locomotor tasks could be the inability to increase the reflex stiffness in the paretic Ia afferent pathway. Functionally, modulation of the reflex gain via changes in presynaptic inhibitory mechanism acts as a protective mechanism to prevent the saturation of afferent volleys and overdrive of motoneuron activation [2, 60], such that the motoneuron pool can effectively receive modulatory information from sensory afferent feedback, and thus play an important role in sensorimotor processes for postural adjustment. Based on our observations in this study, this mechanism is impaired post-stroke.

### Limitations and future studies

We recognize that our findings using this mechanically-constrained paradigm to study locomotion has limitations when generalizing the results to overground walking. A line of research conducted in our lab has used this paradigm to study cortical activity and spinal reflex activity during constrained locomotor behavior with much success [61–63], and this setup was successful in elucidating the postural control component from locomotor output [33]. Future studies will extend this pedaling work by using a robotic device to examine the effects of postural loads on locomotor control under comparable mechanical output.

The non-seated postural loaded pedaling condition was a challenging task for individuals post-stroke. To ensure that all subjects were able to complete this more challenging task, we recruited stroke-impaired individuals of relatively high functional levels. However, despite the higher functional status, the post-stroke participants were still impaired in motor function and force output [33]. To accommodate for this, the target force levels for each participant was set at a percentage of the individual’s maximum force output.

In order to detect changes in H-reflex amplitude during the loading phase of the locomotor task, we chose the point in the pedaling cycle at which the H-reflex is at its greatest amplitude [64], in the middle of the downstroke. Exploration of the full cycle of reflex activity could possibly reveal the modulation characteristics post-stroke.

## Conclusion

Successful execution of upright locomotion is a complex task that requires coordinated interaction between controllers for locomotion and posture. Using a mechanically-constrained pedaling paradigm, we examined the role of the Ia afferent loop in the middle of the downstroke phase, under postural loaded versus non-postural loaded conditions, in the stroke-impaired versus the non-impaired nervous system. Our findings indicate that chronically post-stroke, despite relatively high functional status, ability to coordinate descending postural control commands with the spinal Ia afferent reflex pathway remains impaired. This impairment potentially underlies the interaction between postural control and walking deficits in people with post-stroke hemiplegia. Our results provide the evidence basis to incorporate postural unloading into locomotor or balance training for stroke patients who are otherwise unable to conduct locomotor or balance training under full body loads.
